# A case of novel DYT6 dystonia variant with serious complications after deep brain stimulation therapy: a case report

**DOI:** 10.1186/s12883-022-02871-3

**Published:** 2022-09-12

**Authors:** M. Grofik, M. Cibulka, J. Olekšáková, M. Turčanová Koprušáková, T. Galanda, J. Necpál, P. Jungová, E. Kurča, J Winkelmann, M. Zech, R. Jech

**Affiliations:** 1 Department of Neurology, Jessenius Faculty of Medicine in Martin, Comenius University in Bratislava and University Hospital Martin, Martin, Slovakia; 2 Biomedical Centre Martin, Jessenius Faculty of Medicine in Martin, Comenius University in Bratislava, Bratislava, Slovakia; 3 Department of Neurosurgery, Slovak Medical University and Roosevelt Hospital, Banska Bystrica, Slovakia; 4 Department of Neurology, Zvolen Hospital, Zvolen, Slovakia; 5 Department of Molecular and Biochemical Genetics - Centre of Rare Genetic Diseases, Faculty of Medicine & Comenius University, University Hospital Bratislava, Bratislava, Slovakia; 6 Institute of Neurogenomics, Helmholtz Centrum, Munich, Germany; 7 Institute of Human Genetics, Technical University of Munich, Munich, Germany; 8 Department of Neurology, Charles University, 1st Faculty of Medicine and General University Hospital, Prague, Czech Republic

**Keywords:** DYT6, Dystonia, Deep brain stimulation, Hemorrhage, Seizures

## Abstract

**Background:**

DYT6 dystonia belongs to a group of isolated, genetically determined, generalized dystonia associated with mutations in the *THAP1* gene.

**Case presentation:**

We present the case of a young patient with DYT6 dystonia associated with a newly discovered c14G>A (p.Cys5Tyr) mutation in the *THAP1* gene. We describe the clinical phenotype of this new mutation, effect of pallidal deep brain stimulation (DBS), which was accompanied by two rare postimplantation complications: an early intracerebral hemorrhage and delayed epileptic seizures. Among the published case reports of patients with DYT6 dystonia, the mentioned complications have not been described so far.

**Conclusions:**

DBS in the case of DYT6 dystonia is a challenge to thoroughly consider possible therapeutic benefits and potential risks associated with surgery. Genetic heterogeneity of the disease may also play an important role in predicting the development of the clinical phenotype as well as the effect of treatment including DBS. Therefore, it is beneficial to analyze the genetic and clinical relationships of DYT6 dystonia.

## Background

DYT6 dystonia is associated with mutations in the *THAP1* gene. This gene encodes the DNA-binding transcription factor THAP1, which acts as a nuclear proapoptotic protein, but its exact role is still unknown [1]. DYT6 dystonia is characterized by autosomal dominant inheritance with incomplete penetration. It manifests in childhood, adolescence, or early adulthood (4-20 years) and usually begins in the craniocervical area in approximately half of the patients, with subsequent spread to the limbs. In the rest of the patients, the opposite propagation pattern is observed, with the symptoms starting in the hands [2]. Treatment includes anticholinergic therapy and local denervation with botulinum neurotoxin (BoNT). In cases of drug-resistant dystonia with severe functional impairment, DBS of the globus pallidus internum (GPi) can be considered [3].

## Case presentation

We report the case of a 15-year-old boy with a normal perinatal history and psychomotor development. No neurological disease was observed in the family.

The patient's first difficulties began at the age of 9 years and manifested as graphospasm of the right upper limb. Therefore, the patient gradually started to write with his left upper limb, but here too, at the age of 11 years, he developed graphospasm, which made it impossible for him to write with left upper limb. The patient took the tests orally, and when written tests were required, he took them on a computer keyboard. At the age of 11 years, the dystonia extended to the distal parts of the lower limbs, where it was only mild degree and did not limit the patient in normal activities (such as walking). At the age of 15, cervical dystonia (CD) with the rotation of the head to the left began to develop. At the age of 18, oromandibular dystonia (OMD) was associated with a dominant impairment of the mimic muscles, mainly orbicularis oris (manifested as lip puffiness) and tongue (characterized by tongue protrusion). Mild jaw opening was also present, without spasmodic dysphonia and dysphagia.

The patient had an early score of 55 points on the Burke-Fahn-Marsden Dystonia Rating Scale (BFMDS). The patient's greatest handicap was speech impairment resulting from OMD and facial manifestations (resulting from dysfunction of the orbicularis oris muscle). The second severe handicap was CD. The limb dystonia was subjectively assessed by the patient as not severe and not limiting. Objectively, however, it was a dystonia of severe degree (inability to write, play a musical instrument) but with preserved ability to work with a mobile phone and PC. The patient had permanent resting dystonic postures of hands.

**Fig. 1:**
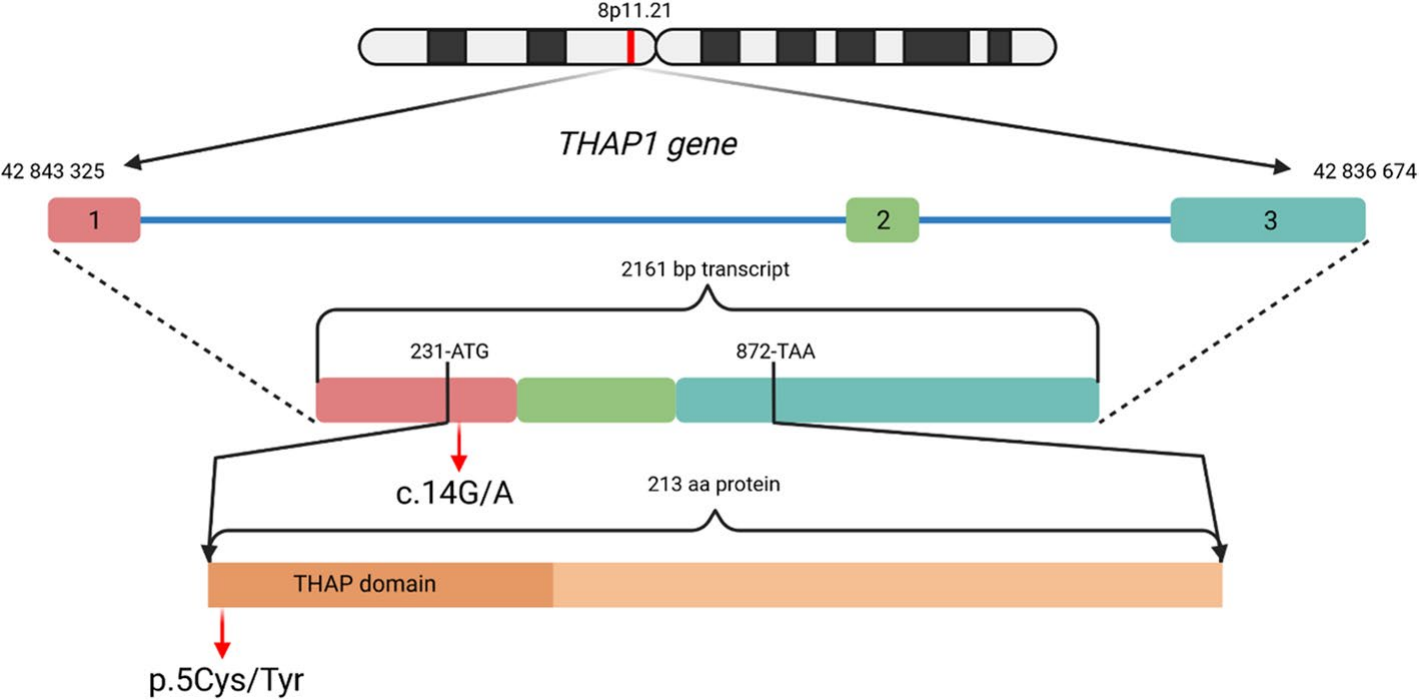
Structure of *THAP1* gene with the detected heterozygous missense variant c.14G>A (p.Cys5Tyr) in our proband (Source: co-author Michal Cibulka; created in BioRender). 1,2,3- exons, bp – base pair, aa- amino acid, ATG- start codon (methionine), TAA- stop codon, c14G/A – substitution of adenine for guanine in the position 14 of coding sequence, p.5Cys/Tyr – substitution of tyrosine for cysteine in the position 5 of polypeptide chain

Apart from the dystonic manifestations described above, no other central and peripheral nervous systems manifestations were present. The patient’s cognitive status and brain MRI was normal. We carried out genetic testing by means of whole exome sequencing performed on HiSeq4000 platform (Illumina, CA, USA). A novel heterozygous missense variant c.14G>A (p.Cys5Tyr) was detected in the *THAP1* gene (Fig. 1). Our finding was first reported by Zech and colleagues among results of multicenter whole-exome sequencing study focused on finding of monogenic causes of dystonia [4].

The presence of this mutation was also determined in the patient's sister (with focal dystonia of the upper limbs arising at the age of 11 years) and their mother (without dystonia).

The patient was treated with biperiden (6 mg per day) for one year; however without any observable effect on dystonia. Higher doses were accompanied by adverse effects, especially dry mouth which made it difficult for the patient to pronounce. The patient received repeated applications of abobotulinum toxin A for CD in the right sternocleidomastoid and the left splenius capitis muscle at a total dose of 500-1,000 IU every three to four months for a period of three years. Its effect, however, gradually weakened and the treatment was stopped. OMD was treated with abobotulinum toxin A to the orbicularis oris (40 IU), depressor anguli oris (10 IU), genioglossus (40 IU) and platysma (40 IU), with an average frequency of application of every three months. This treatment is of mild effect and the patient is still being treated with it.

**Fig. 2:**
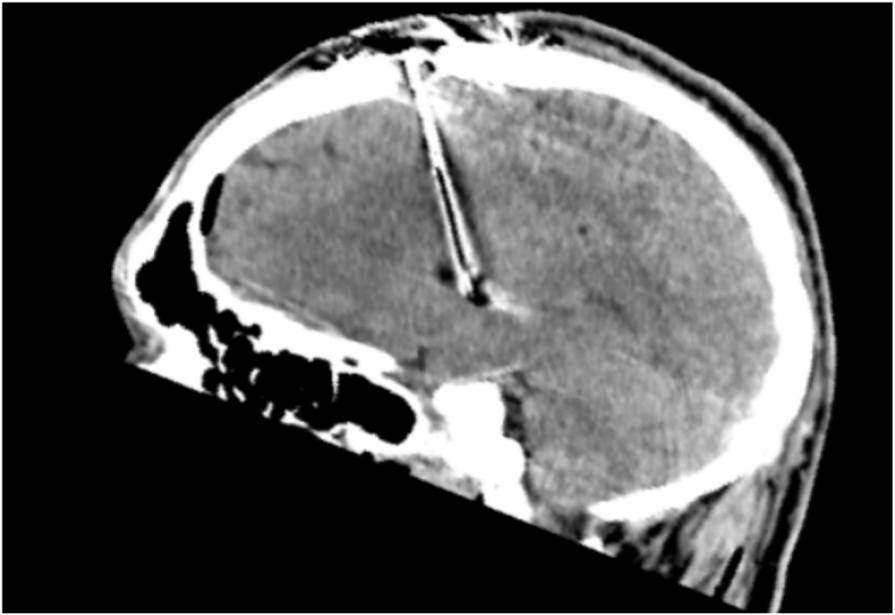
CT scan of brain (immediately after surgery, sagittal view). Without bleeding along the left electrode. The image quality is partially affected by artifacts from the electrode (electrode – black color; artifact around the electrode - white color)

**Fig. 3:**
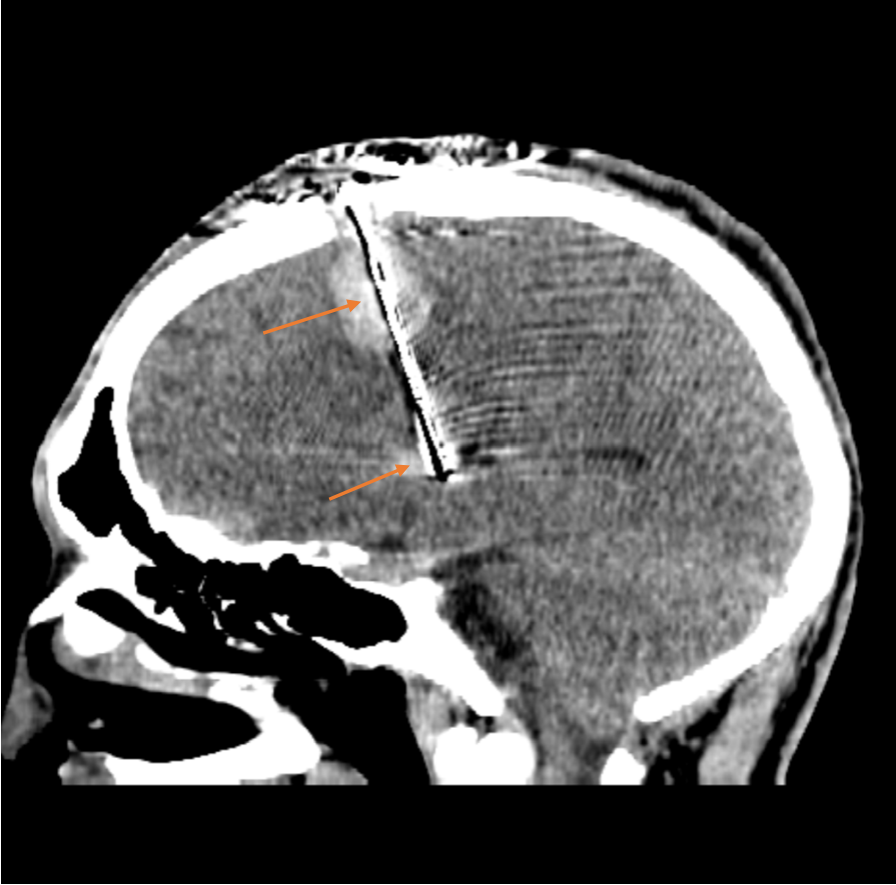
CT scan of brain (fourth postoperative day, sagittal view). Bleeding along the left electrode with maximum in the cortical area, at the site of insertion of the electrode, and in the globus pallidum internum, at the end of the electrode (red arrows). The image quality is partially affected by artifacts from the electrode (electrode – black color; artifact around the electrode - white color)

**Fig. 4:**
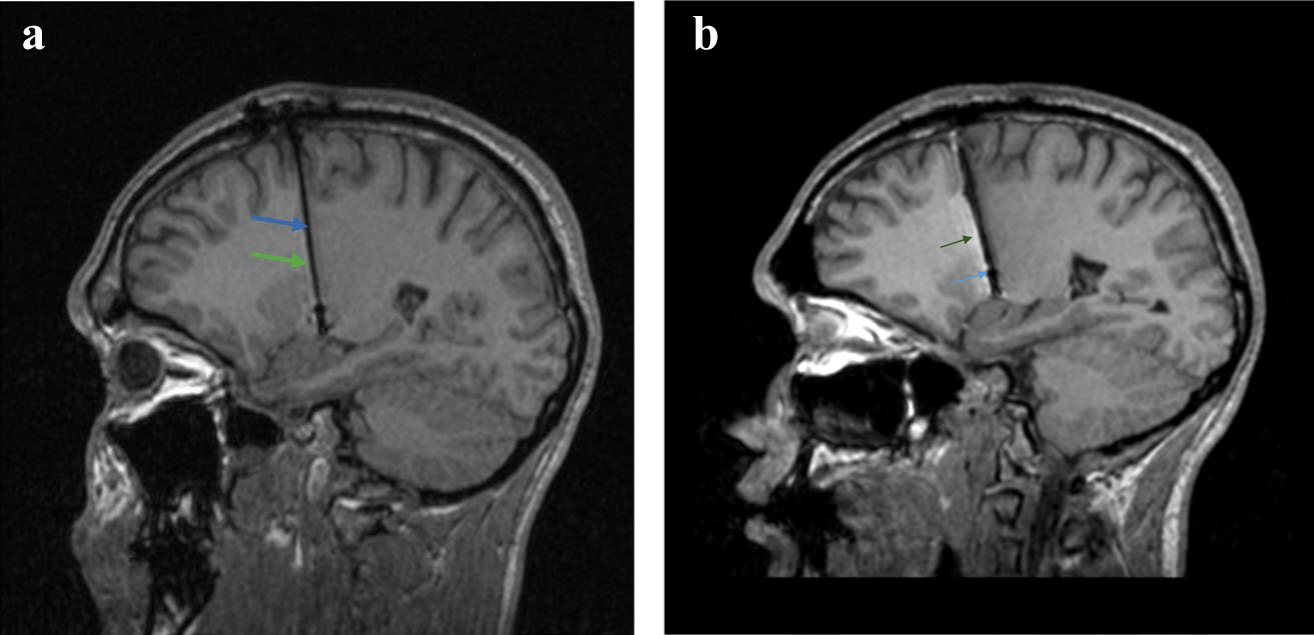
MRI images of the brain- T1, sagittal section, one year after electrode implantation. Normal postoperative findings. **a** right electrode (blue arrow - electrode, green arrow - artifact – thin white border around the electrode). **b** Left electrode (blue arrow - electrode, green arrow - artifact - distinctive white border around the electrode)

**Fig. 5:**
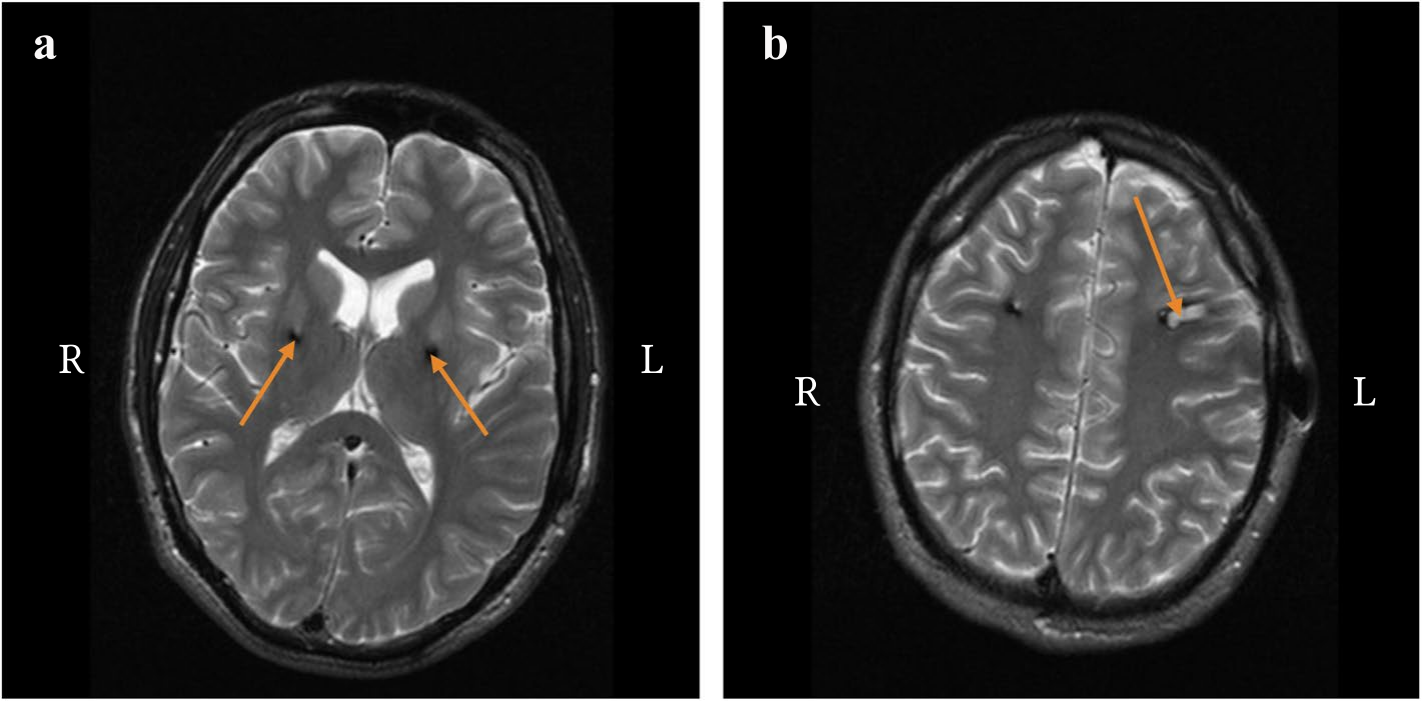
MR brain images, T2, transversal section, one year after electrode implantation. **a** Arrows point to the area of globus pallidum internum - normal postoperative findings at the electrode insertion site. **b** The arrow points to the posthemorrhagic pseudocyst in cortico-subcortical area on the left

The patient was indicated for DBS and was subsequently implanted with electrodes into the bilateral GPi (electrodes 3389, stimulator Activa PC, Medtronic). At the time of the surgery, the patient was 20 years old. The operation as such was without any complications. The CT scans of the brain performed immediately after surgery were within normal limits (Fig. 2). On the fourth postoperative day there was a sudden onset of mild expressive aphasia with no paresis of the limbs. The CT scan showed intracerebral hemorrhage along the left electrode, extending from the cortical area at the site of electrode insertion to the end of the electrode in the GPi (Fig. 3). The patient had no proven vascular malformation on preoperative MRI of the brain and cerebral vessels. As a result of this complication, DBS was not initiated until two months after implantation when the speech disorder was completely corrected and the regression of the hematoma was verified during a control MRI examination of the brain. After six months of DBS treatment, we observed a 30% improvement in BFMDS. The patient had an improvement in dystonia in the upper and lower limbs and a slight improvement in CD but OMD remained intact (speech disorder, protrusion of the tongue, facial expressions), which handicapped the patient the most. The patient therefore assessed the surgical outcome as unsatisfactory. Clinical evaluation was performed with DBS stimulation parameters: monopolar setup, frequency 130 Hz, pulse duration 180 ms, stimulation localization –distal contacts in both of the electrodes (type 3389, Medtronic, MN), stimulation intensity 2.9 V on both sides. Subsequently, bipolar setup was tested with different stimulation electrode contacts, high- and low-frequency stimulation (40, 130, 180 Hz), varying pulse durations (60, 90, 120 and 180 ms) and stimulation intensities (1.2 – 3.5 V) and none of these settings achieved the desired effect on OMD and only a minimal effect on CD was noted. OMD partially responded to abobotulinum toxin A injections. Due to the insufficient effectiveness of DBS, we considered the possibility of posthemorrhagic structural changes of the tissue in the vicinity of the stimulation contacts of the left electrode and we performed a control MRI of the brain one year after implantation. The examination showed a posthemorrhagic cortico-subcortical pseudocyst located out of electrode contacts. (Figs. 4 and 5).

**Fig. 6:**
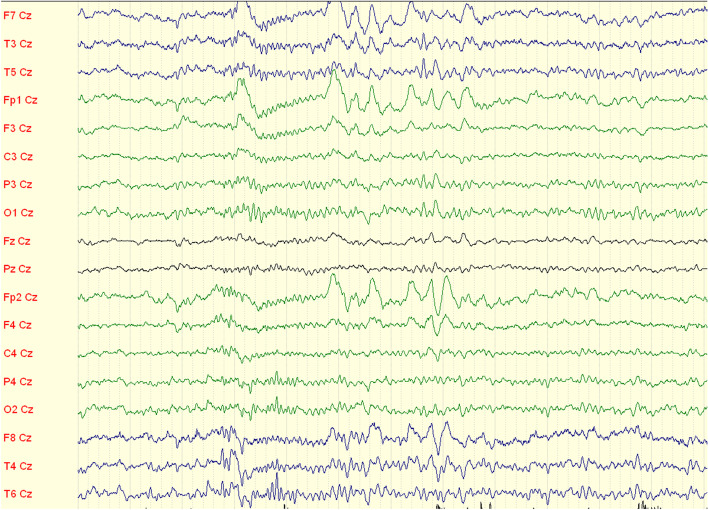
EEG recording - episodes of bifrontal rhythmic delta waves with left-side amplitude accentuation and abortive spike-wave complexes (1 unit=1 sec)

Two years after DBS implantation, the patient experienced the first generalized epileptic tonic-clonic seizure with an unknown onset. The control MRI scans of the brain were without any changes. An EEG showed episodes of bifrontal rhythmic delta waves with left-side amplitude accentuation and abortive spike-wave complexes (Fig. 6). One year later, the patient experienced a second generalized tonic-clonic seizure and subsequently treatment with levetiracetam was started at a dose of 1000 mg daily.

The patient is nowadays 23 years old (three years after DBS implantation) and his BFMDS score is 55 points (the same as preoperative). There is a severe degree of CD approximately in the same extent as before the implantation, severe OMD which has slightly progressed compared to the preimplantation condition and very mild dystonia at the distal region of the upper and lower limbs which have improved with DBS. The patient is treated with levetiracetam and abobotulinum toxin A injections into the oromandibular muscles with a partial effect. Speech impairment is still a major determinant of the patient’s quality of life. Cognitive functions are normal.

## Discussion and conclusions

### Genotype-phenotype correlation

In our case report, we describe the case of DYT6 dystonia, with the detected heterozygous missense variantc.14G>A (p.Cys5Tyr) in the*THAP1* gene. The stated variant is located in an evolutionarily highly conserved region. Experiments indicate that the substitution of cysteine for alanine at the studied position leads to a loss of the ability of THAP1 to bind to DNA [5]. The cysteine at position five of the polypeptide chain participates in the structure of a zinc finger-type motif [6]. It is thus highly likely that the detected cysteine substitution for tyrosine leads to a disruption of the structure of the aforementioned binding motif. According to prediction programs, it is most likely a pathogenic mutation that causes the disease DYT6 in our proband.

Heterogeneous genetic background of DYT6 in terms of numerous likely pathogenic variants detected in *THAP1* might be responsible for a very variable clinical phenotype as well as therapy outcome including DBS. This is in direct opposition with DYT1 dystonia, which is usually caused by single common GAG deletion, resulting in relatively uniform clinical phenotype and predictable response on DBS therapy [7].

### DBS treatment

Postoperatively, there was a significant improvement in dystonia on the limbs; however, no improvement in the craniocervical area. Inconsistent results of DBS treatment have been observed with DYT6 dystonia, mostly showing slight improvement [8, 9], although there are studies describing a significant improvement [10]. The average rate of improvement in motor skills after DBS ranged from 15% to 55% [11]. In most cases, dystonia in the limbs, neck and torso was alleviated, while OMD remained unaffected. The reasons for poorer and more variable DBS response in DYT6 dystonia are still not fully understood but may in part relate to prominent bulbar involvement which is a body region usually less responsive to DBS. We cannot exclude the influence of DBS-related complications, but we did not find convincing fibroproductive changes in the vicinity of the stimulating electrode contacts on MRI, as well as the impedance values when adjusting the stimulation parameters were within normal intervals. Another possibility is genetic heterogeneity in *THAP1* gene where many different pathogenic mutations have been described, whereas DYT1 dystonia is usually caused by a single common GAG deletion [7]. Despite the aforementioned limitations of DBS therapy in relation to the effect on OMD, we proceeded with this therapy because of the expected good effect on limb and cervical dystonia. Subjectively, the patient did not consider these difficulties to be the most significant, but objectively, a severe degree of progressive dystonia was present. Moreover, the patient was in favor of a surgical treatment, which he perceived as a chance to alleviate symptoms refractory to previous therapeutic approaches.

### DBS implantation–related complications

#### Intracerebral hemorrhage

On the fourth postoperative day, the patient experienced bleeding along the left electrode. Such complication has arisen only once in our experience (incidence 1.2% per lead). According to the literature, the incidence of intracerebral hemorrhage is 0.6 to 3.5% per lead. They are mostly small and asymptomatic, do not require surgery and they appear most often during a short time interval after implantation (3.7%) rather than during the surgery (1.1%) [12]. Various factors like age, sex, cognitive status, and target areas (GPi, subthalamic nucleus, ventral intermediate nucleus of the thalamus) were analyzed for the cause of hemorrhage but only number of trajectories for microelectrode recording proved to be statistically significant [13]. We performed microrecording from five trajectories in our patient. A possible explanation for delayed hemorrhage may be damage to small blood vessels or bleeding into venous ischemia with damage to cortical and dural veins. Implantation may result in venous outflow obstruction with subsequent venous hypertension and congestion, leading to hemorrhage and cerebral edema [12].

#### Epilepsy

Two years after implantation, the patient experienced the first generalized epileptic tonic-clonic seizure with unknown onset, which recurred one year later. We hypothesize that it was a focal epileptic seizure that generalized into a bilateral tonic-clonic seizure originating from the left frontal area from an area near the posthemorrhagic residuum. In a recent study, In 814 DBS electrode implantations (645 patients) Atchley et al. [14] reported the incidence of DBS implantation–related seizures as 2.8% per lead (in 3.4% patients). Of all cases with postimplantation-related seizure, epilepsy developed in 17.4% patients postoperatively; the risk of DBS-associated epilepsy was 0.5% per DBS electrode placement and 0.63% per patient. 39.1% implantation-related seizures had have associated postoperative radiographic abnormalities. Multivariate analyses suggested that age at surgery conferred a modest increased risk for postoperative seizures. Sex, primary diagnosis, electrode location and sidedness, and the number of trajectories were not significantly associated with seizures after DBS surgery. Postoperative seizures in 63.6% cases occurred less than 24 hours after placement [14]. The onset of an epileptic seizure 2 years after implantation has not been described in the literature. We also did not encounter the occurrence of postimplantation hemorrhage and epilepsy in the previously published case reports of patients with DYT6. However, we must note that the work analyzing complications of DBS worked with the diagnosis of primary dystonia and thus did not distinguish between different types of dystonia.

DYT6 dystonia is a relatively common primary generalized dystonia with a variable genetic heterogeneity, clinical heterogeneity and variable effect of DBS. This information, along with the possible postoperative complications, must be discussed with the patient prior to the implantation itself.

## Data Availability

All data generated or analyzed during this study are included in this article.

## Abbreviations

AC-PC line: Anterior commissure - posterior commissure line
BFMDS: Burke-Fahn-Marsden Dystonia Rating Scale
BoNT: Botulinum neurotoxin
CD: Cervical Dystonia
CT: Computer Tomography
DBS: Deep Brain Stimulation
DYT: Dystonia
GPi: Globus Pallidum internum
MRI: Magnetic resonance imaging
THAP1: Thanatos-associated Domain-containing Apoptosis-associated Protein 1
OMD: Oromandibular dystonia
