# Correction: A case of novel DYT6 dystonia variant with serious complications after deep brain stimulation therapy: a case report

**DOI:** 10.1186/s12883-022-02931-8

**Published:** 2022-11-03

**Authors:** M. Grofik, M. Cibulka, J. Olekšakova, M. Turčanova Koprušakova, T. Galanda, J. Necpal, P. Jungova, E. Kurča, J. Winkelmann, M. Zech, R. Jech

**Affiliations:** 1 Department of Neurology, Jessenius Faculty of Medicine in Martin, Comenius University in Bratislava and University Hospital Martin, Martin, Slovakia; 2 Biomedical Centre Martin, Jessenius Faculty of Medicine in Martin, Comenius University in Bratislava, Bratislava, Slovakia; 3 Department of Neurosurgery, Slovak Medical University and Roosevelt Hospital, Banska Bystrica, Slovakia; 4 Department of Neurology, Zvolen Hospital, Zvolen, Slovakia; 5 Department of Molecular and Biochemical Genetics - Centre of Rare Genetic Diseases, Faculty of Medicine & Comenius University, University Hospital Bratislava, Bratislava, Slovakia; 6 Institute of Neurogenomics, Helmholtz Centrum, Munich, Germany; 7 Institute of Human Genetics, Technical University of Munich, Munich, Germany; 8 Department of Neurology, Charles University, 1st Faculty of Medicine and General University Hospital, Prague, Czech Republic

**Correction: BMC Neurol 22, 344 (2022)**

10.1186/s12883-022-02871-3

Following publication of the original article [1], the authors reported an error in author group. Author M. Zech should be affiliated to affiliations 6 and 7.

The original article [1] has been updated.
